# Robustness of Cloud Manufacturing System Based on Complex Network and Multi-Agent Simulation

**DOI:** 10.3390/e25010045

**Published:** 2022-12-27

**Authors:** Xin Zheng, Xiaodong Zhang

**Affiliations:** School of Economics and Management, University of Science and Technology Beijing, Beijing 100083, China

**Keywords:** cloud manufacturing, robustness, complex network, multi-agent simulation

## Abstract

Cloud manufacturing systems (CMSs) are networked, distributed and loosely coupled, so they face great uncertainty and risk. This paper combines the complex network model with multi-agent simulation in a novel approach to the robustness analysis of CMSs. Different evaluation metrics are chosen for the two models, and three different robustness attack strategies are proposed. To verify the effectiveness of the proposed method, a case study is then conducted on a cloud manufacturing project of a new energy vehicle. The results show that both the structural and process-based robustness of the system are lowest under the betweenness-based failure mode, indicating that resource nodes with large betweenness are most important to the robustness of the project. Therefore, the cloud manufacturing platform should focus on monitoring and managing these resources so that they can provide stable services. Under the individual server failure mode, system robustness varies greatly depending on the failure behavior of the service provider: Among the five service providers (S1–S5) given in the experimental group, the failure of Server 1 leads to a sharp decline in robustness, while the failure of Server 2 has little impact. This indicates that the CMS can protect its robustness by identifying key servers and strengthening its supervision of them to prevent them from exiting the platform.

## 1. Introduction

In the era of Industry 4.0, advanced information technology such as cloud computing and the Internet of Things has brought profound changes to the manufacturing industry. Li et al. [1] conceptualized a new service-oriented networked manufacturing model known as “cloud manufacturing”, which aggregates manufacturing resources and capabilities into the cloud platform, and fully realizes the sharing of manufacturing resources and capabilities through service integration [2].

This concept has received much attention from academia and enterprises, and a great amount of research has already been carried out on various aspects of cloud manufacturing, including its hierarchical structure [3,4], typical features [5], key technologies [5,6,7,8,9,10,11,12], operation modes [13,14,15,16] and service portfolio scheduling [17,18,19,20].

The cloud manufacturing system (CMS) is networked, distributed and loosely coupled; this creates great uncertainty and interference [21], which is an important issue that the CMS must face and solve. Zhang et al. [22] and Zhu et al. [23] argued that the development of cloud manufacturing is restricted by a lack of trust and security, and blockchain technology provides new ideas for overcoming such restrictions due to its reliability, tamper-proof nature, traceability and high transparency. Further, Laili et al. [24] stated that orders of different tasks affect the CMS. As such, the allocation and scheduling capability of the CMS when facing multiple tasks [25,26,27] is an important component when considering the robustness of the system. Wang et al. [28] studied the impact of service anomalies on the CMS, proposing a dynamic service composition reconfiguration model when anomalies occur. Liang et al. [29] stated that the complex demands of consumers and the changes in the dynamic environment pose challenges to common CMS-based scheduling algorithms. They proposed deep reinforcement learning to enable the system to overcome these challenges through continuous training and learning. In their study on the typical problems of cloud manufacturing, Tao et al. [6] proposed that uncertain factors exist within the cloud manufacturing life cycle, which can be classified into five categories: uncertainty of tasks, uncertainty of resource services, uncertainty of quality of service (QoS), uncertainty of the correlation between resource services and uncertainty of other factors. The CMS faces complex and diverse types of uncertain interference. This includes not only common interference found in the traditional manufacturing field, but also unique, uncertain factors found in the cloud manufacturing field [30].

It is of great practical significance for the implementation and deployment of cloud manufacturing projects to (a) accurately identify the impact of uncertain factors on cloud manufacturing, (b) explore the robustness level of the cloud manufacturing process (CMP) under different disturbances and attacks, and (c) further improve the stability and invulnerability of the system. However, existing research on the robustness of the CMS is currently lacking. The complex network approach is commonly used in the study of network robustness, where the entities in the system are abstracted as “nodes”, the relationships between entities are “connected edges”, and relevant topological parameters (e.g., degree, betweenness, agglomeration coefficient) reflect the structural characteristics of the system as a whole. Commonly used robustness measures include the relative size of connected subgraphs, average distance and network efficiency, among others. To explore the robustness and vulnerability problems of traditional production processes, Li et al. [31] constructed a complex multi-task directed weighted network using (a) nodes to represent equipment, personnel and departments in the production plant, and (b) connected edges to represent logistical and process-based relationships. They took network connectivity and the maximum connected subgraph as the robustness measurement indexes, comparing the robustness of the workshop network under both random and selective interference. Shi et al. [32] and Shi et al. [33] used sensitivity analysis to explore the effects of factors such as the number of nodes, number of interconnected links, interconnection mode, scaling index and load capacity on the interdependent supply network robustness under random and intentional disruptions. The robustness index was selected with consideration given to the heterogeneity of different nodes, and the connected sub-network with the largest size including all kinds of nodes (LACS) is proposed to replace the traditional connected sub-network with the largest size (LCS). Fan et al. [34] proposed a two-layer maintenance support service network composed of an undirected network layer and a directed entity layer, comparing the changes in network robustness under three different partnership construction strategies. Moghaddam and Deshmukh [35] studied the robustness of cyber-physical production systems using a complex network method for the scenarios of cascading and non-cascading disruptions. Based on the characteristics of these two disruption modes, different formulae for measuring robustness metrics were given.

The above research shows that the complex network method can satisfactorily reflect the structural characteristics of the CMS. It also has a wide range of applications (e.g., in manufacturing networks and supply chain networks) because it can be used to obtain the change of robustness index under different network attack strategies. However, because this method abstracts the entities and inter-entity relationships structurally, it is then difficult to reflect the logical judgments and dynamic temporal relationships between entities.

In contrast, simulation models can accurately describe the behavioral interactions and temporal relationships of the entities of manufacturing systems. Simulation research on the CMS is also a popular topic in current research. For example, Zhao et al. [2] designed and implemented an agent-based cloud manufacturing simulation platform. They provided a high-level encapsulation of services in the cloud platform, including a five-layer (i.e., data, low tool, management, upper tool and application) architecture of the cloud platform. In Zhang et al. [36], typical intelligent manufacturing simulation technologies were analyzed from three aspects: manufacturing unit simulation, manufacturing integration simulation and manufacturing intelligent simulation. Various other perspectives have also been studied, such as cloud service entity packaging [37,38], selection and scheduling [39,40,41], and trust and security issues [23]. However, despite its importance, simulation-based research on the robustness of the CMS remains rare.

This study proposes a robustness analysis method that combines complex networks with multi-agent simulation to analyze the robustness of the CMS from two perspectives: a static structure and dynamic process. The remainder of the paper is organized as follows. In Section 2, a multi-agent simulation model of the CMP is constructed. Here, the behavioral characteristics and models of several key agents in the CMP are given, and QoS is proposed as a robustness measure. Section 3 explores the robustness of the static topology of the cloud manufacturing network (CMN). Here, the complex network model of cloud manufacturing resources is established through both the order–task relationship and the task–resource relationship, and network efficiency and the largest connected subgraph are proposed as robustness measures. In Section 4, the robustness attack strategies are designed, where a degree-based resource failure mode (ID), betweenness-based resource failure mode (IB) and individual server-based resource failure mode are proposed. In Section 5, a case study of a cloud manufacturing project is presented, and its robustness is studied under different failure modes by combining the multi-agent simulation software Anylogic and Python 3.0 tools. Section 6 provides the conclusions and prospects of this paper.

## 2. CMP Model and Robustness Measurement Index Based on Multi-Agent Simulation

### 2.1. Multi-Agent Simulation Model Construction

**Figure 1:**
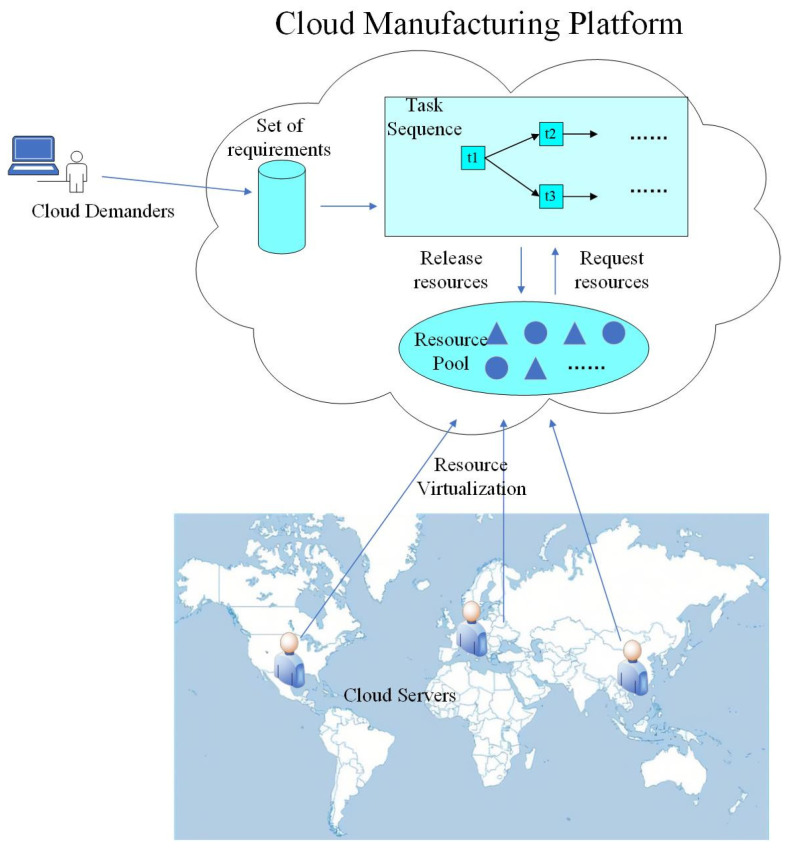
Schematic diagram of cloud manufacturing process.

The CMS includes the cloud platform, cloud task, cloud resource, cloud message, order and other types of subjects, as well as two types of user role: cloud service providers and cloud demanders [3]. As shown in Figure 1, the CMP [1] broadly includes the following:

(1) Cloud service providers unify various types of manufacturing equipment resources and manufacturing capability resources into the cloud platform, depositing them into the cloud resource pool through information transformation, resource sensing, resource access, unified modeling of cloud services and other technology. This bypasses the limitations of space and distance by enabling resources that are originally distributed across the world to be centrally managed and shared.
(2) Cloud demanders submit service requirements (i.e., orders) to the cloud platform through terminal devices. Orders from multiple cloud demanders are uniformly stored in the cloud demand set, waiting to be processed.
(3) According to the service route of the order to be processed, the cloud platform integrates and adapts different cloud tasks to form orderly and stable cloud task sequences.
(4) When the cloud demand set is not empty, the platform imports each order into the corresponding cloud task sequence, in turn, to carry out cloud manufacturing services. When the cloud tasks are being processed, the corresponding resources are requested from the resource pool according to the task type. Resources in an idle state change to a busy state after being requested. After the task is completed, the resource is released and returned to an idle state.

The CMS contains multiple entity types, and various forms of information transmission and behavior interaction occur between the same entities and different entities. Therefore, the CMS model can be expressed as follows:

(1) CMS={PA,DA,SA,TA,RA,OA,MA,E}

where PA is the cloud platform agent; DA is the cloud demander agent; SA is the cloud service agent; TA is the cloud task agent; RA is the cloud resource agent; OA is the order agent issued by the DA; MA is the message agent sent to the SA when the TA requests or releases resources; and E is the external environment of information transmission and behavior interaction among entities.

#### 2.1.1. Cloud Platform Modeling

The cloud platform is the center of the CMS. The cloud demander sends orders to the cloud platform, and the platform assigns these orders to the corresponding tasks. Throughout the CMP, the platform records any processing successes and failures, and at the end of the service cycle, relevant performance indicators (e.g., service time, cost, reliability, order completion rate) are calculated. Additionally, the platform carries out a variety of roles, such as model parameter initialization and experimental parameter adjustment. The cloud platform agent can be expressed as follows:

(2) $$PA=<tobeProcessedOrderList,finishedOrderList,\;failedOrderList,attackNum,Func_{ini},Func_{allocationOrders},Func_{settingFaultStatus},\;Func_{calcuQos},Func_{outputNetwork}>$$

where tobeProcessedOrderList stores orders sent by each cloud demander that are to be processed; finishedOrderList records successfully completed orders; failedOrderList records failed orders; attackNum is the number of failed resources; $Func_{ini}$ is used to initialize model parameters; $Func_{allocationOrders}$ assigns orders to their respective corresponding cloud tasks for processing; $Func_{settingFaultStatus}$ sets the specified resource of the specified server to the failure state according to the node failure mode; $Func_{calcuQos}$ counts data related to service time, cost, reliability and the order completion rate at the end of simulation, then integrates these to calculate the QoS index; and $Func_{outputNetwork}$ sorts the order–task relationship and task–resource relationship of the processed orders into a node-list form and outputs it, to be used to construct the complex network model.

#### 2.1.2. Cloud Resource Modeling

Cloud resources are the virtual resources formed by integrating the manufacturing equipment resources and manufacturing capability resources of service providers into the cloud resource pool through information transformation, resource access, cloud service unified modeling and other technology. The main function of the cloud resource agent is to cooperate with cloud tasks to complete the processing of cloud orders:

(3) RA=<ID,produceLevel,busy,broken,owner,price>

where ID is the unique identifier number of the resource; produceLevel is the productivity level of the resource, which is defined as an integer from 1–10; busy indicates whether the resource is in a busy state; broken indicates whether the resource is faulty; owner specifies which cloud server the resource belongs to; and price represents the cost of the resource, which is randomly generated with normal distribution during model initialization.

#### 2.1.3. Cloud Task Modeling

The construction of the cloud task agent is key to cloud manufacturing simulation modeling. It covers not only the processing path of all order types (e.g., serial, parallel, hybrid path) but also the behavior interaction and information transfer between the cloud server agent and the cloud resource agent. In addition, the cloud task agent formulates (a) the selection mechanism of the optimal service provider, (b) various statistical data, such as service cycle and cost, and (c) cloud task and cloud resource node information. To achieve this, existing process modeling library components are adapted accordingly. The cloud task agent can be expressed as follows:

(4) $$TA=<ID,owner\underline{}Orders,pre\underline{}taskList,\;after\underline{}taskList,\;reque\underline{}resourceList,basicWorkingTime,\;currentOrder,Func_{selectBestServer},\;Func_{selectBestResource},\;Func_{recordRouteStamp},\;Func_{recordTaskTime},\;Func_{recordTaskCost},Func_{recordTaskReliability}>$$

where ID is the unique identification number of the task; $owner\underline{}Orders$ specifies which type of order processing path the task belongs to; $pre\underline{}taskList$ and $after\underline{}taskList$ specify the pre-order task and post-order task, respectively; $reque\underline{}resourceList$ specifies the type of resource requested by the task; basicWorkingTime specifies the standard time for completing the task; currentOrder represents the order currently being processed; $Func_{selectBestServer}$ determines the optimal server based on resource price, logistics, distance and other factors; $Func_{selectBestResource}$ determines the optimal resource; $Func_{recordRouteStamp}$ records the order–task relationship and task–resource relationship for completed orders; and $Func_{recordTaskTime}$, $Func_{recordTaskCost}$ and $Func_{recordTaskReliability}$ record the service time, service cost and service reliability of the current task, respectively.

**Figure 2:**
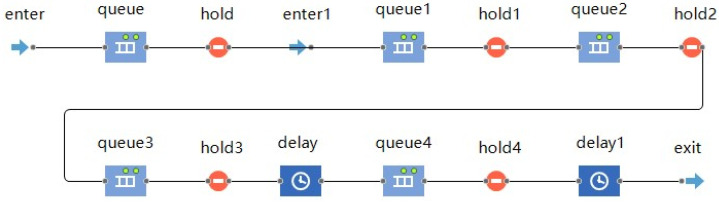
Internal process of cloud task agent.

Figure 2 shows the detailed CMP simulation inside the cloud task agent, which is realized by editing and adapting the existing component codes from Anylogic’s process modeling library. The details of this process are as follows:

(1) The order is imported into the internal process of the cloud task through the *enter* component. If the current task is first in the task sequence, the order is directly assigned by the cloud platform; otherwise, the order is assigned by the preceding task after its completion (e.g., task 2 orders are assigned from task 1 once task 1 has been completed).
(2) The *queue* component temporarily stores the current order while the following judgments are made: (a) if the current task is first in the task sequence or there is only one task in the preceding task sequence, the *hold* and *hold1* components are simultaneously opened and the current order is entered into *queue2* for subsequent processing; or (b) if there is more than one task in the preceding task sequence, the current order must wait until the orders of all preceding tasks have completed before entering *queue2* for subsequent processing
(3) The *queue1* component merges the information of several branch orders, and the *hold2* component ensures that only one order is entered for subsequent processing at a time. When the current order is completed and exits through *exit*, *hold2* opens again and continues to serve the next order.
(4) The order enters *queue3*, where the task agent selects the optimal server and sends “resource request” information to it. When the optimal service provider accepts the request, the busy attribute corresponding to the optimal resource changes to “true”, and the *hold3* component opens. The order flows through the *delay* component to simulate the cloud manufacturing service. After a certain delay time, the service is completed.
(5) The order enters *queue4* and continues to send the “release resource” message to the optimal server. When the optimal server accepts the message, the busy attribute corresponding to the optimal resource changes to “false”, and the *hold4* component opens. The order flows through the *delay1* component. After a certain delay time, the release of the resource is completed.
(6) The order flows through the *exit* component to complete all its service processes in this task. It then imports the post-order task sequence of this task: (a) if there is only one post-order task, it is directly imported into the *enter* component of the post-order task; (b) if there are multiple post-order tasks, the information of the current order is copied and imported into the *enter* component of the respective post-order tasks; and (c) if there is no post-order task, this signifies that the task is already the final task in the task sequence. As such, the order is added to the set of completed orders, and information such as the service cycle, service cost and route record are counted and output.

#### 2.1.4. Cloud Order Modeling

The orders are submitted to the cloud platform by the cloud demanders through terminal devices. They are then imported to the corresponding cloud tasks according to their respective task routes to complete service processing. The cloud order agent is represented as follows:

(5) OA=<ID,owner,taskList,routeStamp,cost1Accum,cost2Accum,cost3Accum,reliabilityAccum,startTime,finishTime>

where ID is the order’s unique identification number; owner specifies which demander the order is issued by; taskList specifies the complete task path corresponding to the order; routeStamp records the order–task relationship and task–resource relationship corresponding to the order when the order is completed; cost1Accum, cost2Accum and cost3Accum record the request resource cost, logistics cost and release resource cost of the order, respectively; reliabilityAccum records the completion reliability of orders; and startTime and finishTime record the start processing time and completion processing time of the order, respectively.

#### 2.1.5. Cloud Message Modeling

When processing orders, cloud tasks need to send “request resource” information to the cloud server. After the processing is complete, a “release resource” message is sent to the cloud server. Since Anylogic’s built-in message agent cannot carry extra information, this paper encapsulates a messages agent type, which can be expressed as follows:

(6) MA=〈msg,resourceList,owner〉

where msg is the content of the message (i.e., when a resource is requested, the msg’s value is “request”, and when a resource is released, the msg’s value is “release”); resourceList specifies which resources are to be requested and released; and owner indicates which cloud task sent the message.

#### 2.1.6. Cloud Demander Modeling

The cloud demander issues demand orders to the cloud platform to drive the operation of the model. The cloud demander agent can be represented as follows:

(7) $$DA=\langle ID,location,orderList,Func_{sendOrders}\rangle$$

where ID is the cloud demander’s unique identification number; location is the latitude and longitude coordinates of the demander, which is used to initialize the location of the demander in the GIS map; orderList is used to initialize the orders issued by the demander; and $Func_{sendOrders}$ sends the orders of cloud demanders to the cloud platform, where the cloud platform schedules and allocates the orders uniformly.

#### 2.1.7. Cloud Server Modeling

Cloud servers mainly transfer information and interact with cloud tasks. All types of cloud resources are stored in the resource pool of each cloud server. When receiving the “request resource” message, the server finds the corresponding resource in its resource pool and allocates it to the cloud task. When receiving the “release resource” message, the server releases the corresponding cloud resource and puts it back into the cloud resource pool. The cloud server agent can be represented as:

(8) $$SA=<ID,location,resourcePool,dScore,\;pScore,totalScore,Func_{configureResource}>$$

where ID is the unique identification number of the cloud service provider; location is the latitude and longitude coordinates of the server, which is used to initialize the location of the server in the GIS map; resourcePool is used to store the respective virtual resources of the cloud server; dScore, pScore and totalScore are the respective distance score, price score and total score when the cloud task selects the optimal cloud server; and $Func_{configureResource}$ is used to execute and allocate resources when receiving information about cloud tasks. If the message’s content is “release resource”, the server finds the corresponding resource and changes its busy attribute to “false”. If the message’s content is “request resource”, the corresponding resource is judged as follows: (a) if its value is “true”, this indicates that the resource is faulty and the cloud task cannot be completed; as such, the order requested for processing is classified as failed; or (b) if the value is “false”, this indicates that the resource is not faulty. Here, its busy attribute is judged as follows: (i) if the busy attribute is “false”, the processing of the cloud order can be started, and (ii) if the busy attribute is “true”, this indicates that the resource is being invoked by other tasks and it needs to wait until the other task is completed.

### 2.2. Robustness Measurement Index Based on Multi-Agent Simulation

Based on the multi-agent model and order task sequence stated in Section 2.1, the dynamic simulation of the CMP can be realized. At the end of the simulation, the order completion time, logistics transportation distance, resource occupation and other data can be output to evaluate the performance. QoS is commonly used to evaluate the CMP. As such, based on the combination of relevant literature and the simulation output data, this paper comprehensively evaluates the QoS value from four aspects: service time, service cost, service reliability and order completion rate [20,42,43]. The calculation formulae of these four indicators are as follows:

(1) Service time

This is the sum of the completion times of all orders within the simulation cycle, which can be expressed as

(9) $$T=\overset{m}{\underset{j=1}{\sum }}t_{j}$$

where m is the total number of orders; j = 1, 2,…, *m* is the *j*th order in the order sequence; and $t_{j}$ is the completion time of the *j*th order, which can be obtained by the simulation results.

(2) Service cost

This total cloud service cost is calculated from three aspects: the cloud resource service fee, logistics service fee and cloud resource release fee, which can be expressed as

(10) $$C=\overset{m}{\underset{j=1}{\sum }}\overset{n_{j}}{\underset{i=1}{\sum }}t_{i,j}^{serving}*p_{i,j}^{resource}+\overset{m}{\underset{j=1}{\sum }}\overset{n_{j}}{\underset{i=1}{\sum }}d_{i,j}*c^{logistic}+\overset{m}{\underset{j=1}{\sum }}\overset{n_{j}}{\underset{i=1}{\sum }}t_{i,j}^{releasing}*p^{release}$$

where m is the total number of orders; n is the number of tasks corresponding to each order; j = 1, 2,… *m* represents the *j*th order in the order sequence; i = 1,2,… *n* represents the *i*th task in the task sequence; $t_{i,j}^{serving}$ is the cloud service time of the ith task in the *j*th order; $p_{i,j}^{resource}$ is the service cost per unit time of the resource corresponding to the task; $d_{i,j}$ is the logistics distance corresponding to the task; $c^{logistic}$ is the logistics cost per unit distance; $t_{i,j}^{releasing}$ is the release time of the cloud resources for the task; and $p^{release}$ is the cost per unit time of releasing resources.

(3) Service reliability

Service reliability is a multiplicative index [42], which can be expressed as

(11) $$rel=\frac{\sum_{j=1}^{m}\prod_{i=1}^{n_{j}}r_{i,j}}{m}$$

where $r_{i,j}$ is the service reliability of the *i*-th task in the *j*-th order, which is given in the *reliabilityAccum* attribute of the order agent.

(4) Order completion rate

The order completion rate is the ratio of the number of completed orders within the simulation cycle to the total number of orders planned to be completed:

(12) $$ofr=\frac{N_{1}}{N_{1}+N_{2}}$$

where $N_{1}$ is the number of orders completed within the simulation cycle and $N_{2}$ is the number of orders that failed to be completed.

In addition, the index values need to be standardized to consider the different index dimensions. A series of robustness experiments will be carried out later in this paper, so range standardization is carried out with the index values of each experiment as samples. The calculation formulae are as follows:

(13) $$nt=\frac{T_{k}-T_{min}}{T_{max}-T_{min}}$$

(14) $$nc=\frac{C_{k}-C_{min}}{C_{max}-C_{min}}$$

where $T_{k}$ and $C_{k}$ are the respective service time and service cost of the *k*-th experiment; $T_{min}$ and $T_{max}$ are the respective minimum and maximum values of service time; and $C_{min}$ and $C_{max}$ are the respective minimum and maximum values of service cost. The reliability and order completion rate indicators are originally in the range of [0, 1], so there is no need for standardization.

The QoS value can be evaluated by synthesizing the above four dimensions:

(15) $$QoS=\omega_{1}*nt+\omega_{2}*nc+\omega_{3}*rel+\omega_{4}*ofr$$

where $\omega_{1}$, $\omega_{2}$, $\omega_{3}$ and $\omega_{4}$ are the respective weight coefficients of the four indicators, and $\overset{4}{\underset{i=1}{\sum }}\omega_{i}=1$.

## 3. Cloud Manufacturing Network Model and Robustness Measurement Index-Based on Complex Network

### 3.1. Construction of Cloud Manufacturing Complex Network Model

**Figure 3:**
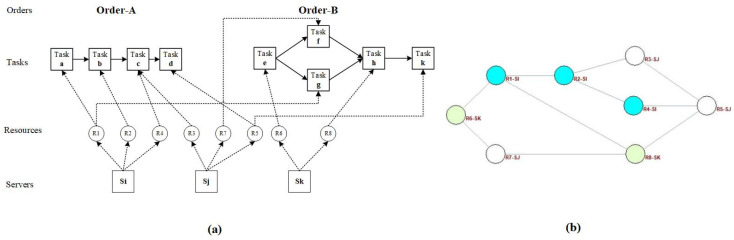
Schematic diagram of (**a**) internal relationship of the cloud manufacturing system (CMS) and (**b**) cloud resource network.

The cloud manufacturing network (CMN) is composed of cloud service resources and the connections between resources. Due to the large number of resources and complex connection relationships, the network can be analyzed using the complex network model. Figure 3a shows the processing task paths of Order-A and Order-B, the resources used by each task in these paths, and the corresponding relationships between resources and servers. If two tasks are connected on a path, the respective resources used by the two tasks are also considered to be connected. Figure 3b shows how the CMN is formed by taking all the resources as network nodes and the connections between resources as connected edges.

### 3.2. Robustness Measurement Index Based on Static Network Topology

It is generally considered that network robustness refers to the degree of network performance retention after the failure of network nodes or edges [44], and the change of the maximum connected subgraph after network node failure can reflect the degree of retention of the network’s structural integrity. As such, the change rate of the maximum connected subgraph’s node number is selected as one of the robustness evaluation indexes in this study:

(16) $$S=\frac{N^{\prime }}{N}$$

where *N′* is the number of nodes in the maximum connected subgraph after the network is attacked, and *N* is the total number of nodes in the original network. In particular, *S* = 0 indicates that the network is in an unconnected state; and *S* = 1 indicates that the network is fully connected, and there is no isolated node.

Additionally, the connections between the nodes change when a network node fails. This, in turn, affects the efficiency of information dissemination in the network. Therefore, network efficiency is used to evaluate the robustness of the network transfer efficiency when nodes are lost. The shorter the distance between two nodes in a network, the faster information can be transferred from one node to another. Based on this, the formula of network efficiency can be defined as:

(17) $$\Phi_{G}=\frac{1}{N\left(N-1\right)}\underset{i\neq j}{\sum }\frac{1}{d_{ij}}$$

where *N* represents the total number of nodes in the network and $d_{ij}$ represents the shortest path between node *i* and node *j*. In particular, *G* = 0 indicates the efficiency of the network is the worst, where the whole network contains isolated nodes, and *G* = 1 indicates that the efficiency of the network is the best, where the information exchange between nodes is smooth.

## 4. Failure Mode Design for Robustness Analysis

The design of failure modes is key to robustness analysis. Based on the characteristics of cloud manufacturing, this paper proposes a topology-based resource failure mode and a server-based resource failure mode.

Topology-based resource failures are further divided into degree-based and betweenness-based resource failures, where (a) node degree (i.e., how closely a resource node is connected to other resource nodes in the CMN) is commonly used to measure a node’s importance, and (b) node betweenness reflects the structural importance of the node [44,45], with a node with high betweenness having greater control over logistics and information flow in the network. The specific topology-based resource failure mode designs are shown in Table 1.

Server-based resource failures fully consider the realistic scenario of cloud manufacturing, where resource nodes involved in cloud manufacturing belong to different cloud servers. The successive failure of resource nodes of the same cloud server can simulate the scenario where the cloud server gradually exits the platform and no longer provides resources. Key cloud servers can be identified by comparing the robustness indexes of different cloud servers after the loss of resources, and focused monitoring and management of these key servers can effectively ensure the robustness of the CMN. The specific server-based resource failure mode designs are shown in Table 2.

## 5. Case Study

### 5.1. Description of Model Parameters

A case study is carried out using the cloud manufacturing project of a new energy vehicle. This project provides life-cycle cloud manufacturing services for new energy vehicles, where the vehicles served are equipped with technology such as electrification and autonomous driving.

The cloud manufacturing project includes 24 order types, 95 cloud tasks (t1–t95) and 72 resource types (r1–r72). The corresponding resource types for each cloud task are shown in Table 3, and the cloud task routes of each order type are shown in Table 4.

A total of 5 cloud servers (S1–S5) participate in the project, with each server providing 72 types of cloud resources. The cloud servers have different pricing of resources, and they are at different distances from the cloud demanders, so they compete for different orders. To distinguish between them, resource r1 of servers S1–S5 are labeled r1-S1, r1-S2, r1-S3, r1-S4 and r1-S5, and so on.

In addition, there are 14 cloud demanders (d1–d14). Each cloud demander submits 24 orders, and the number of orders of each type is 1 (i.e., 1 of each of the 24 order types). The basic information of each cloud service provider and cloud demander is externally imported from Excel, as shown in Table 5.

In this paper, the weight coefficient of QoS is set as $\omega_{1}\;$= 0.35, $\omega_{2}\;$= 0.35, $\omega_{3}\;$= 0.1, $\omega_{4}\;$= 0.2.

### 5.2. Structural Robustness Analysis

**Figure 4:**
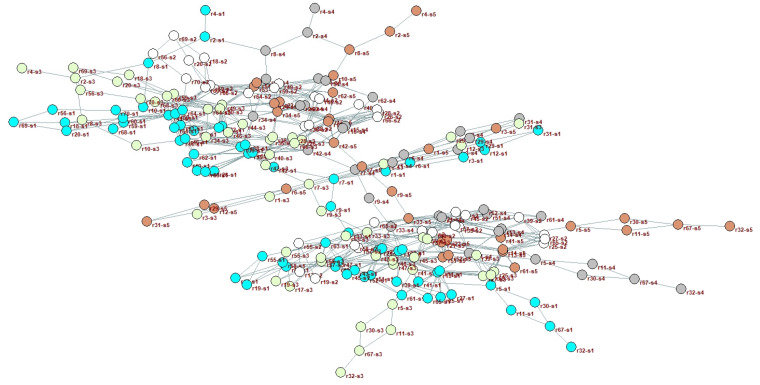
Spatial layout of cloud resource network.

**Figure 5:**
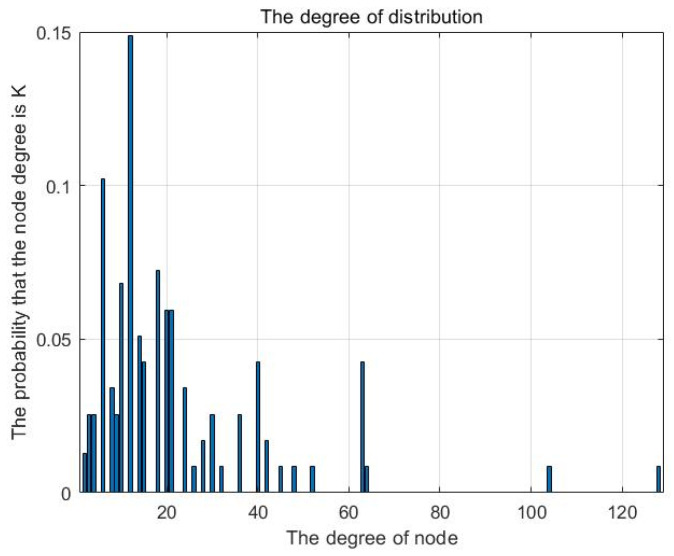
Degree distribution of cloud resource network.

The cloud resource network is obtained according to the network model construction method stated in Section 3.1, as shown in Figure 4. Matlab-2020a software is used to perform data statistics on the network, and both the relevant network topology parameters and the degree distribution are obtained, as shown in Table 6 and Figure 5, respectively.

There are 231 resource nodes in the network, and the distribution of node degrees is seriously uneven. A few nodes occupy the majority of connected edges, indicating that the network has the typical characteristics of a scale-free network. The small density of the network indicates that it is a sparse network, with the nodes with higher degree values tending to connect the nodes with lower degree values.

Next, based on the failure modes designed in Section 4, Python 3.0 is used to simulate and calculate the changes of structural robustness indexes under the different failure modes.

**Figure 6:**
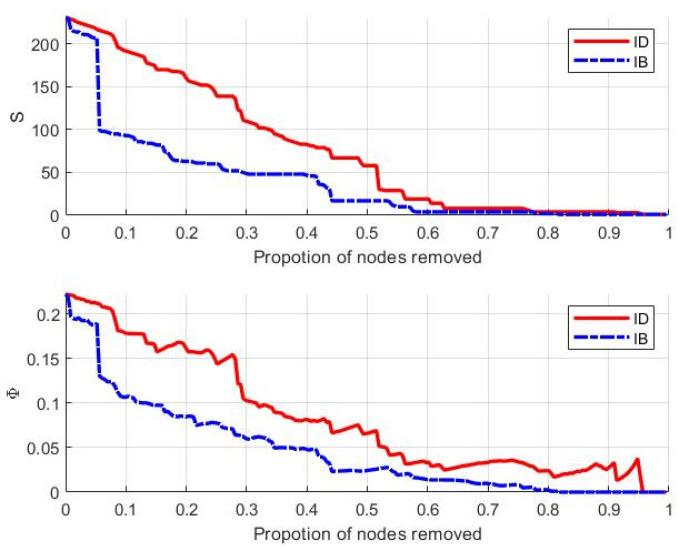
Trend of S and Φ based on topology-based failure mode.

The calculation results based on the topology-based failure mode are shown in Figure 6. This shows that (a) both network efficiency and the largest connected subgraph gradually decrease with the increase of the node failure ratio, and (b) the index value in the IB mode is always lower than that in the ID mode, which indicates that the robustness of the CMN is more fragile in the IB mode. Further, in the IB mode, when the failure ratio is around 0.05, there is a precipitous decline in robustness. In contrast, the overall decline trend of robustness in the ID mode is relatively stable. When the failure ratio reaches 0.4, the maximum connected subgraph in the IB mode drops below 50, and the network efficiency drops below 0.05, which indicates that the network is in a state of collapse. This shows that from the perspective of complex networks, resource nodes with large betweenness are more important to the maintenance of the structural robustness of CMNs. As such, the focus should first be on protecting those nodes with larger betweenness, followed by those nodes with a larger degree.

**Figure 7:**
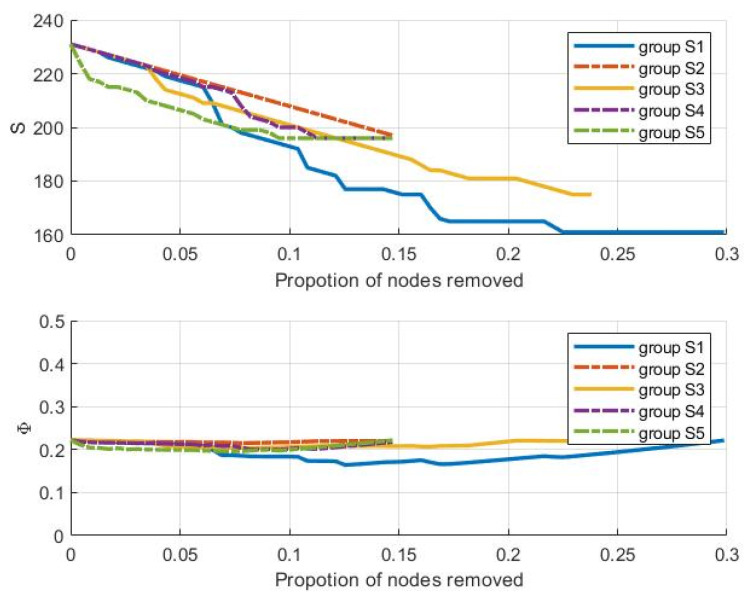
Trend of S and Φ based on cloud server failure mode.

The robustness analysis results under the cloud server failure mode are shown in Figure 7. This shows that (a) the network efficiency value shows a fluctuating trend during the failure of cloud server nodes, and there is no significant decline; however, the maximum connected subgraph decreases rapidly with the increase of the node failure ratio, which indicates that the maximum connected subgraph is more sensitive to the failure of the cloud server; and (b) the maximum connected subgraph of S1 not only decreases the most out of the 5 servers (from 231 to 161), but it also shows a significant decline when the node failure ratio is in the [0.05, 0.1] interval. Further, the maximum connected subgraph of S3 decreases from 231 to 175, and the respective maximum connected subgraphs of S2, S4 and S5 are all above 195 after being attacked. From the perspective of the complex network, the key cloud servers are selected as S1 followed by S3. These servers should be monitored and managed to protect the structural robustness of the CMN.

### 5.3. Process Robustness Analysis

**Figure 8:**
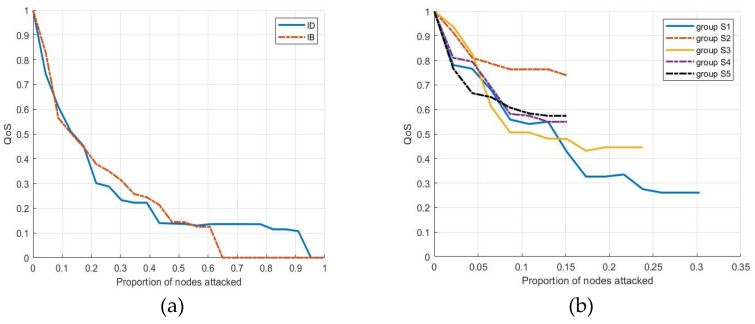
Trend of QoS change based on (**a**) topology-based failure mode and (**b**) cloud server failure mode.

In addition to the process robustness measures and failure modes, this paper uses multi-agent simulation software Anylogic and Python 3.0 to explore the variations of QoS under the different failure modes, as shown in Figure 8.

As shown in Figure 8a, QoS rapidly decreases with the increase of the node failure ratio in both the IB and ID modes. For both modes, the QoS values drop below 0.25 when the node failure ratio is about 0.4. However, in the IB mode, all cloud orders fail to be processed when the node failure ratio is about 0.65, whereas in the ID mode, this occurs only when the node failure ratio is about 0.95. This shows that the robustness of the CMP is more fragile in the IB mode. From the perspective of multi-agent simulation, nodes with large betweenness are more important for maintaining the robustness of the dynamic processes of cloud manufacturing.

As shown in Figure 8b, the QoS index of S1 decreases the most (from 1 to less than 0.3), followed by S3 (from 1 to 0.44). Further, the QoS indexes of S2, S4 and S5 are all above 0.5 after being attacked, which indicates that the resource failure of these servers has little impact on the robustness of the CMP. From the perspective of multi-agent simulation, S1 and S3 are the key cloud servers, which is consistent with the evaluation results from the complex network perspective stated in Section 5.2.

## 6. Conclusions

This paper proposes a novel approach for the robustness analysis of the CMS, combining complex network analysis with multi-agent simulation, which extends the robustness analysis object from the CMN to the CMP. First, a multi-agent simulation model is constructed. The behavioral characteristics and models of several key agents in the CMP are detailed, and QoS is proposed as a robustness measure. Second, a complex network model of cloud manufacturing resources is established through both the order–task relationship and the task–resource relationship to investigate the robustness of the static topology of the CMN. For this, network efficiency and the maximum connected subgraph are proposed as robustness measures. Three attack strategies are designed, which are resource failure modes based on the degree, betweenness and individual server. To verify the feasibility and effectiveness of the proposed method, a case study is then conducted on a cloud manufacturing project of a new energy vehicle. The results show that the robustness of the system (both for the CMN and the CMP) is lowest under the betweenness-based resource failure mode. This indicates that resource nodes with large betweenness are most important to the structural robustness and process robustness of the project. As such, the CMP should focus on monitoring and managing these cloud manufacturing resources so that they can provide stable services. Under the server-based failure mode, the robustness of the system varies greatly depending on the failure behavior of the service provider (e.g., the failure of S1 leads to a sharp decline in robustness, but the failure of S2 has little impact). This indicates that the CMS can protect its robustness by identifying key servers and strengthening its supervision of them to prevent them from exiting the platform.

This paper primarily focuses on how various failure modes affect the performance of the CMS, and it proposes related robustness analysis methods and protection measures. Future research based on this established complex network and multi-agent simulation model will involve the design of corresponding recovery strategies and elasticity measures of the CMS. Simulation research will also be carried out to provide a quantitative and dynamic decision basis for the improvement of the robustness of the cloud manufacturing platform.

## Figures and Tables

**Table 1:** Design of topology-based resource failure modes.

	Failure Mode Description	Failure Mode Calculation Process
Topology-based resource failure modes	Initial node degree loss (ID)	Sort the resource nodes in the initial network by degree, from largest to smallest. Remove one node at a time, and repeat n times until all nodes in the network are removed.
	Initial node betweenness loss (IB)	Sort the resource nodes in the initial network by betweenness, from largest to smallest. Remove one node at a time, and repeat n times until all nodes in the network are removed.

Note: The removal of nodes is performed differently in the complex network model and the multi-agent model: (a) the complex network model is performed by deleting the corresponding resource nodes and all connected edges on the nodes, and (b) the multi-agent model is represented by setting the corresponding resource agent to a “fault” state, that is, where the resource cannot provide services.

**Table 2:** Design of server-based resource failure modes.

	Failure Mode Description	Failure Mode Calculation Process
Server-based resource failure modes	Successive failure of Server-1′s node (group S1)	Select the resource nodes belonging to server S1 in the CMN. Remove one node at a time, and repeat n times until all resource nodes belonging to server S1 in the network are removed.
	Successive failure of Server-2′s node (group S2)	Select the resource nodes belonging to server S2 in the CMN. Remove one node at a time, and repeat n times until all resource nodes belonging to server S2 are removed.
	⋯⋯	⋯⋯
	Successive failure of Server-n’s node (group Sn)	Select the resource nodes belonging to server Sn in the CMN. Remove one node at a time, and repeat n times until all resource nodes belonging to server Sn are removed.

**Table 3:** Correspondence between tasks and resources.

Task	Resource	Task	Resource	Task	Resource	Task	Resource
t1	(r1)	t2	(r3)	t3	(r2)	t4	(r4)
t5	(r11, r30)	t6	(r5)	t7	(r12, r29)	t8	(r6)
t9	(r12, r29)	t10	(r31)	t11	(r11, r30)	t12	(r67)
t13	(r32)	t14	(r1)	t15	(r7)	t16	(r1)
t17	(r2)	t18	(r8)	t19	(r2)	t20	(r5)
t21	(r5)	t22	(r41)	t23	(r6)	t24	(r6)
t25	(r71)	t26	(r42)	t27	(r33)	t28	(r9)
t29	(r9)	t30	(r34)	t31	(r10)	t32	(r10)
t33	(r9)	t34	(r7)	t35	(r9)	t36	(r7)
t37	(r10)	t38	(r8)	t39	(r10)	t40	(r8)
t41	(r13, r14)	t42	(r61)	t43	(r21, r23)	t44	(r51, r52)
t45	(r15, r16)	t46	(r62)	t47	(r22, r24)	t48	(r53, r54)
t49	(r21, r23)	t50	(r47, r48)	t51	(r47, r48)	t52	(r33)
t53	(r35, r37)	t54	(r63)	t55	(r43, r45)	t56	(r22, r24)
t57	(r49, r50)	t58	(r49, r50)	t59	(r34)	t60	(r36, r38)
t61	(r64)	t62	(r44, r46)	t63	(r35, r37)	t64	(r41)
t65	(r41)	t66	(r39)	t67	(r25, r27)	t68	(r65)
t69	(r13, r14)	t70	(r36, r38)	t71	(r42)	t72	(r42)
t73	(r40)	t74	(r26, r28)	t75	(r66)	t76	(r15, r16)
t77	(r47, r48)	t78	(r51, r52)	t79	(r57, r58)	t80	(r47, r48)
t81	(r49, r50)	t82	(r53, r54)	t83	(r68)	t84	(r59, r60)
t85	(r49, r50)	t86	(r57, r58)	t87	(r17, r19)	t88	(r63)
t89	(r55)	t90	(r59, r60)	t91	(r18, r20)	t92	(r69)
t93	(r64)	t94	(r70)	t95	(r56)		

**Table 4:** Correspondence between orders and cloud service routes.

Orde Type	Cloud Service Route	Order Type	Cloud Service Route
Order 11	t1 → t2	Order 12	t3 → t4
Order 13	t5 → t6	Order 14	t7 → t8
Order 15	t9 → t10	Order 16	t11 → t12 → t13
Order 21	t14 → t15 → t16	Order 22	t17 → t18 → t19
Order 23	t20 → t21 → t22	Order 24	t23 → t24 → t25 → t26
Order 25	t27 → t28 → t29	Order 26	t30 → t31 → t32
Order 31	t33 → t34 → t35 → t36	Order 32	t37 → t38 → t39 → t40
Order 33	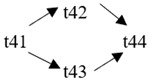	Order 34	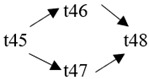
Order 41	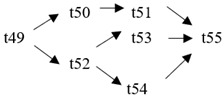	Order 42	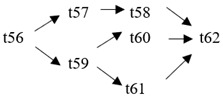
Order 43	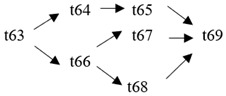	Order 44	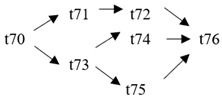
Order 51	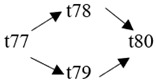	Order 52	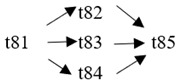
Order 53	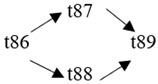	Order 54	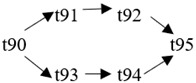

**Table 5:** Attributes of cloud servers and cloud demanders.

ID	City	Location (Latitude, Longitude)	ID	City	Location (Latitude, Longitude)
S1	Beijing	(39.91, 116.41)	d5	Jinan	(36.4, 117)
S2	Shanghai	(31.21, 121.43)	d6	Lanzhou	(36.03, 103.73)
S3	Chengdu	(30.66, 104.06)	d7	Wulumuqi	(43.76, 87.68)
S4	Hangzhou	(30.26, 120.2)	d8	Changsha	(28.21, 113)
S5	Shenzhen	(22.61, 114.06)	d9	Nanchang	(28.68, 115.9)
			d10	Fuzhou	(26.08, 119.3)
d1	HaErbin	(45.75, 126.63)	d11	Nanning	(22.48, 108.19)
d2	ShenYang	(41.8, 123.38)	d12	Lasa	(29.6, 91)
d3	Baotou	(40.39, 109.49)	d13	Lianyungang	(34.36, 119.1)
d4	Tianjin	(39.13, 117.2)	d14	Hefei	(31.52, 117.17)

**Table 6:** Topological parameters of cloud resource network.

Topological Parameter	Number of Nodes	Average Degree	Density	Average Path Length
Cloud resource network	231	21.208	0.087	4.231

Notes: (1) The average degree is the average of the degree of all nodes in the network. (2) The density of a network can be expressed as the ratio of the actual number of edges to the maximum possible number of edges in the network. (3) The average path length is the average of the distance between any two nodes in the network.

## Data Availability

The used and analyzed datasets during the present study are available from the corresponding author on reasonable request.
